# Wild Mammals as Sentinels for West Nile Virus Circulation: Evidence from Serbia

**DOI:** 10.3390/pathogens14111167

**Published:** 2025-11-15

**Authors:** Ljubiša Veljović, Milan Paunović, Dimitrije Glišić, Sofija Šolaja, Zorana Zurovac Sapundžić, Jelena Maletić, Bojan Milovanović, Vesna Milićević

**Affiliations:** 1 Institute of Veterinary Medicine of Serbia, Janisa Janulisa 14, 11000 Belgrade, Serbia; dimitrije.glisic@nivs.rs (D.G.); sofija.solaja@nivs.rs (S.Š.); zorana.zurovac@nivs.rs (Z.Z.S.); jelena.maletic@nivs.rs (J.M.); bojan.milovanovic@nivs.rs (B.M.); vesna.milicevic@nivs.rs (V.M.); 2 MM Consulting Agency, Rade Končara 7, 11107 Belgrade, Serbia; mm.consulting.belgrade@gmail.com

**Keywords:** West Nile fever, seroprevalence, ELISA, nutria, deer, wild boar, jackals, Serbia

## Abstract

West Nile fever is a mosquito-borne zoonotic disease caused by West Nile virus (WNV), maintained in an enzootic cycle between avian hosts and *Culex* mosquitoes. While birds are the principal reservoirs, WNV also infects a wide range of mammals, including humans, horses, and wildlife species. In this study, we assessed WNV seroprevalence in wild ungulates, wild boars, golden jackals, and the invasive rodent nutria in Serbia. A total of 522 serum samples from wild animals were tested. Antibodies against WNV were detected across all tested species, with seroprevalence rates of 37% in wild boars, 11.9% in nutrias, 32.4% in golden jackals, 50.6% in red deer, and 9.1% in roe deer. Detection of antibodies in both adults and juveniles provides evidence of recent transmission during the study period. These findings confirm widespread circulation of WNV in Serbian wildlife and suggest that wild ungulates, carnivores, and invasive rodents may serve as useful sentinel species for monitoring WNV prevalence and geographic spread in natural ecosystems.

## 1. Introduction

West Nile fever is an arthropod-borne zoonotic viral disease that is transmitted from birds to humans and other susceptible animals by mosquitoes [1]. The causative agent of the disease is West Nile Virus (WNV), which belongs to the genus *Orthoflavivirus*, family *Flaviviridae*, and is a member of the Japanese encephalitis serocomplex [2]. The virus was first described in Africa in 1938 and has since then spread across Europe, the Middle East and Asia, causing major human epidemics [3]. The virus is maintained in an enzootic cycle between avian hosts and mosquito vectors, especially those belonging to the *Culex* genus [4]. Climatic factors play a critical role in shaping the distribution of WNV and thus the endemicity of WNF. Precipitation patterns and fluctuations in the levels of rivers, lakes, ponds, and wetlands strongly influence mosquito abundance while simultaneously altering wild bird habitats. As wild birds are natural reservoirs of WNV, these climatic conditions further govern the long-term maintenance of the virus in nature [1]. Several bird species are capable of developing neutralising antibodies against WNV, providing long-lasting protection across multiple transmission seasons. This has been documented in species such as the house sparrow (*Passer domesticus*) [5] and various raptors.

Many non-avian species are reported as susceptible to the WNV infection. At least 100 mammal species develop an immune response after exposure to WNV [6,7]. West Nile antibodies have been detected in wild mammals and primates [8], with wild boars and ruminants proposed as valuable sentinels of circulation [9,10]. The invasive nutria (*Myocastor coypus*) has also emerged as a potential reservoir, posing additional ecological risks [11]. Humans and horses are generally considered dead-end hosts, as they do not develop a viremia high enough to infect mosquitoes [12].

Since its emergence in North America in 2001, WNV has evolved into a major One Health concern, driving recurrent outbreaks in humans and animals across Europe and beyond. The 2010 Greek outbreak, subsequent cases in Italy, and the record 2018 European epidemic underscored its public health impact [13,14,15]. In Serbia, human infections were first confirmed in 2012, with *Culex pipiens* identified as the principal vector [4,16]. While most infections remain asymptomatic, a fraction progress to severe neuroinvasive disease with significant mortality [17].

The Western Balkans is recognised as a critical crossroads for the introduction and dissemination of West Nile virus (WNV) lineages originating from both Western–Central and Southern Europe, with frequent mutations reported at virulence-associated sites [18]. In this context, continuous, systematic, and broad surveillance remains essential. To improve the accuracy of WNV prevalence estimates, the testing of multiple sentinel species within shared habitats is recommended. Accordingly, this study investigated the seroprevalence of WNV in selected wild game and invasive species hunted in Serbia.

## 2. Materials and Methods

**Figure 1:**
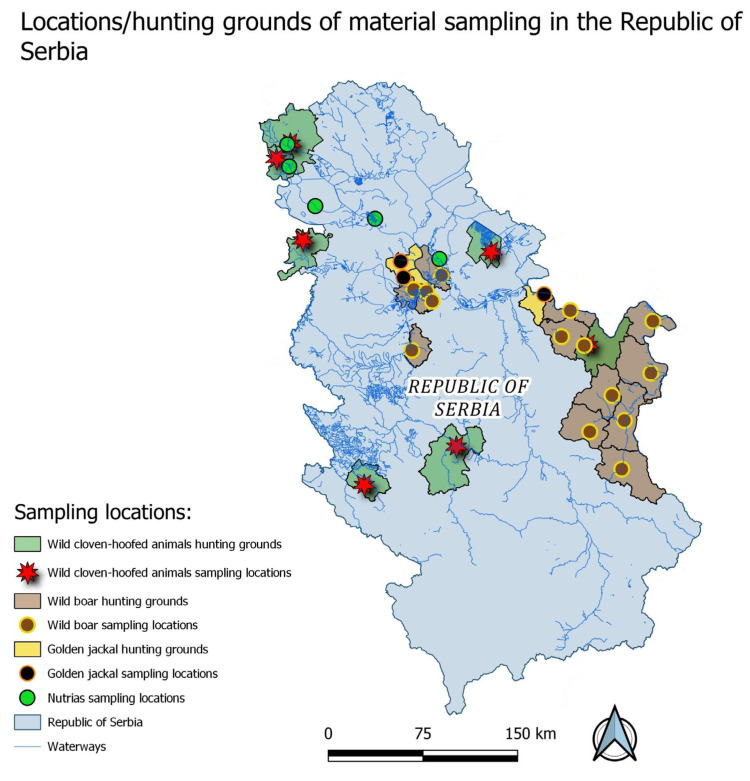
Location of hunting grounds of material sampling in the Republic of Serbia.

A total of 522 serum samples from wild animals were analysed, including wild boar (*Sus scrofa*), roe deer (*Capreolus capreolus*), red deer (*Cervus elaphus*), golden jackal (*Canis aureus*), and nutria (*Myocastor coypus*). Samples were collected across Serbia (Figure 1) during the winter of 2023/2024 winter months (January, February and early March) from 19 hunting grounds, while nutrias were obtained along riverbanks. All animals were obtained during routine culling programmes aimed at population control; therefore, no ethical approval was required. The location of the hunting grounds of material sampling in the Republic of Serbia is presented in Figure 1.

The hunting grounds from which the samples of wild ungulates were obtained are located in central, eastern, and in the western part of Serbia. The hunting grounds from which golden jackals were sampled are located in eastern Serbia and in the vicinity of Belgrade, while the hunting grounds from which wild boars were sampled are mostly located in eastern and central Serbia. Nutria samples were collected along the banks of the Danube River. Figure was produced using QGIS Version 3.28.3 Firence [QGIS.org (2024). QGIS Geographic Information System. Open Source Geospatial Foundation Project, “http://qgis.org”].

Age classification of sampled animals was performed to distinguish juvenile and adult categories for epidemiological analysis. Classification was based on species-specific morphological and dental criteria. In nutria, body weight and length (≤3 kg or ≤45 cm indicating juveniles) were used. In wild boar, tooth eruption and wear patterns served as the main indicators (juveniles < 1 year, adults > 1 year). For jackals, body size, tooth wear, and reproductive maturity were considered (juveniles < 1 year, adults > 1 year). In wild ruminants, age determination relied on dentition, body size, and antler development (juveniles < 1 year, adults > 1 year) (Table 1).

A non-probability convenience sampling method was used, selecting animals based on accessibility and availability. Sampling bias was reduced because all specimens came from animals culled during the same hunting season. Blood samples were obtained either directly from the thoracic cavity of freshly hunted animals or during veterinary health inspections. After spontaneous coagulation at room temperature, sera were separated by centrifugation (Eppendorf, Germany) at 2000× *g* for 10 min, decanted, and stored frozen until further analysis.

To detect specific anti-WNV antibodies, a multi-species blocking enzyme-linked immunosorbent assay (ELISA) [19] was used, following the manufacturer’s recommendations. According to the producer validation data, the diagnostic sensitivity (Se) of this kit is 100% and the diagnostic specificity (Sp) is 98.4%. Prevalence, Lower Confidence Limits (CL) and Upper Confidence Limits were calculated by use of [20], according to Wilson’s confidence interval method and regarding a confidence level of 95% with desired precision of +/−0.05. Ref. [20] was also used for the calculation of true prevalence for an imperfect test with known Se and Sp of the test. To compare statistical significance between groups by species and age categories, we used the “Chi-squared test for contingency table from original data” in Epitools. We based our calculations on the desired 0.95 level of confidence.

## 3. Results

A total of 522 serum samples from five wild mammal species were tested, of which 165 were seropositive, resulting in an overall seroprevalence of 31.6% (TP = 30.5%; 95% CI: 0.27–0.35) (Table 1). Among the examined species, the highest seroprevalence was detected in red deer, with 50.6% positive samples (TP = 48.8%; 95% CI: 0.42–0.58), followed by wild boars with 37.0% (TP = 36%; 95% CI: 0.28–0.47) and golden jackals with 32.4% (TP = 31.3%; 95% CI: 0.22–0.44). Lower seroprevalence values were observed in nutrias (11.9%, TP = 10.5%; 95% CI: 0.07–0.20) and roe deer (8.6%, TP = 7.2%; 95% CI: 0.03–0.15).

Seropositivity was more frequent among adults (136 positive; 33.7%) compared to juveniles (29 positive; 24.4%), indicating a clear age-related difference in exposure. Among juveniles, seroprevalence was generally lower, with the exception of juvenile golden jackals showing relatively high positivity (45.5%) compared to other species (Table 1). For statistical significance of positive and negative samples between groups by species, we obtained a Chi-square of 65.15 with *p* value < 0.0001, while for statistical significance between age categories, we obtained a Chi-square of 43.82, and *p* value between <0.0001. The results of such high chi-square values as well as low *p* values indicate the existence of statistically significant differences between groups, as well as between age categories of samples (Table 2).

## 4. Discussion

In this study, we evaluated the seroprevalence of WNV in various species of wild ungulates, golden jackals, and nutrias collected during a single hunting season using ELISA. It should be noted that the ELISA kit used cannot distinguish between antibodies against WNV and Usutu virus; therefore, the seroprevalence results should be interpreted with caution, as antibodies to the Usutu virus were not excluded by the virus neutralisation test. The presence of WNV-specific IgG in older animals generally indicates past infection [21]. Because the blocking ELISA test cannot differentiate between IgM and IgG or between recent and past exposure, seroprevalence results in juveniles must also be interpreted carefully, as their antibodies may be of maternal origin. However, seropositivity in young, immature individuals born after the previous vector season provides more reliable evidence of recent viral circulation.

As expected, adults exhibited higher seroprevalence than juveniles across all tested species, reflecting cumulative exposure to infected mosquito bites [16]. Nevertheless, the detection of seropositive juveniles among wild boars, red deer, roe deer, jackals, and nutrias demonstrates active transmission during the sampling year.

Epidemiological data on WNV seroprevalence in nutrias are scarce. Previous studies have reported antibodies in other rodent species, including the fox squirrel (*Sciurus niger*), eastern grey squirrel (*Sciurus carolinensis*), groundhog (*Marmota monax*), yellow-necked field mouse (*Apodemus flavicollis*), and bank vole (*Myodes glareolus*) [6]. In our survey, nutrias showed a seroprevalence of 11.9%, with antibodies also detected in two immature individuals, supporting evidence of recent seasonal infections in this invasive species. The detection of WNV antibodies in nutria was expected, given that their typical wetland habitats are also favoured by mosquito vectors. Thus, WNV seroprevalence in this species may offer valuable insights into viral distribution beyond human settlements—particularly in coastal zones, stagnant waters, lakes, and riverbanks, which represent unpopulated habitats shared with vectors. Interestingly, the observed seroprevalence was lower than expected, which may be related to environmental or host-specific factors such as the nutria’s dense double coat composed of soft underfur and long guard hairs.

Across Europe, WNV seroprevalence in wild ungulates has generally been low: 0% in wild boars from Poland [9], 4–10% in several other surveys [22,23,24], and as low as 4.04% in wild boars and 0.23% in red deer across multiple bioregions [6]. Even the higher prevalence values reported in Spain—21.9–26.6% in wild boars and 17.6–21.4% in red deer [25]—remain below our findings. Similarly, studies by [26,27] reported 4–6% prevalence in wild boars, red deer, and roe deer.

WNV prevalence in deer and wild boar was of particular interest because these two species are the most common in Serbian hunting grounds. They share similar habitats—dense forests with high humidity and limited sunlight—which provide favourable conditions for mosquito vectors. In contrast to previous reports, our study revealed markedly higher true seroprevalence values: 36% in wild boars and 50.6% in red deer, while roe deer showed comparable rates (7.2%). Comparable results were reported by [28], who found 49.1% positivity in red deer and 7.8% in roe deer populations. The slightly higher prevalence observed in our study suggests more intense and persistent WNV circulation in the investigated regions, indicating ongoing viral activity exceeding levels previously documented in European wild ungulate populations.

The high prevalence of WNV antibodies in young jackals may be related to their geographic origin. Jackals were sampled in hunting grounds along the Danube River in northeastern Serbia, as well as in areas connecting the Sava and Danube rivers. Riverbanks and wetlands provide ideal habitats for high mosquito densities, likely overlapping with the areas where young jackals were sampled. However, the total number of juvenile jackals examined (*n* = 11) was relatively small.

Taken together, our findings demonstrate substantially higher WNV seroprevalence in wild boars and red deer compared to previous European data, where prevalence typically ranged between 0% and 10% and only occasionally exceeded 20% [22,25,26]. While roe deer results (8.6%) were consistent with earlier reports, the elevated seroprevalence observed in red deer (50.6%) and wild boars (37%) underscores intensified WNV circulation in Serbian ecosystems. These differences may reflect regional ecological conditions, increased vector activity, or unique host–vector interactions within the study areas. Collectively, our results highlight the value of wild ungulates and other wildlife species as sentinels for WNV monitoring in endemic regions.

The detection of WNV antibodies in nutria—an aquatic species closely associated with wet habitats—provides valuable data on viral presence and vector populations along rivers and lakes. In contrast, WNV seropositivity in golden jackals indicates virus circulation and vector activity throughout the hunting grounds from which they were sampled, many of which are located near Serbia’s the largest rivers, the Danube and the Sava.

The locations of the hunting grounds are not populated places, but with the banks of the rivers, they represent an attractive tourist destination and potential risk to the human population. A large number of nature-loving visitors, as well as bathers on the banks of rivers, are probably more exposed to infection because these areas overlap with the favourite ecosystems of the vector.

## 5. Conclusions

This study provides new evidence of widespread WNV circulation in Serbian wildlife, with seroprevalence levels in wild boars and red deer exceeding those reported elsewhere in Europe. The detection of antibodies in both adult and juvenile individuals confirms recent viral activity and ongoing transmission. Our results emphasise the need for continuous, multi-species surveillance to better understand the epidemiology of WNV and to strengthen One Health approaches for early warning, risk assessment, and control of mosquito-borne diseases. This especially applies to red deer and wild boar, where the seroprevalence showed a significantly higher level compared to other examined species of wild mammals.

## Figures and Tables

**Table 1:** Samples distribution species and age category.

Species	Number of Samples	Adults	Juvenile
Wild boar	100	53	47
Nutria	101	87	14
Golden jackal	68	57	11
Red deer	172	137	35
Roe deer	81	69	12
Total No of samples	522	403	119

**Table 2:** Seroprevalence of WNV antibodies in wild mammals by species and age group.

Species	Total Tested	Total Positive (%)	TP (%)	95% CI (Lower–Upper)	Adults Tested	Adults Positive (%)	Juveniles Tested	Juveniles Positive (%)
Wild boar	100	37 (37.0)	36.0	0.28–0.47	53	23 (43.4)	47	14 (29.8)
Nutria	101	12 (11.9)	10.5	0.07–0.20	87	10 (11.5)	14	2 (14.3)
Golden jackal	68	22 (32.4)	31.3	0.22–0.44	57	17 (29.8)	11	5 (45.5)
Red deer	172	87 (50.6)	48.8	0.42–0.58	137	81 (59.1)	35	6 (17.1)
Roe deer	81	7 (8.6)	7.2	0.03–0.15	69	5 (7.2)	12	2 (16.7)
Total	522	165 (31.6)	30.5	0.27–0.35	403	136 (33.7)	119	29 (24.4)

## Data Availability

The raw data supporting the conclusions of this article will be made available by the authors on request.

## Abbreviations

The following abbreviations are used in this manuscript:

WNV	West Nile Virus
ELISA	Enzyme-linked immunosorbent Assay
CL	Confidence Limits
Se	Diagnostic sensitivity
Sp	Diagnostic specificity
TP	True Prevalence
